# A PEG-mediated protoplast transformation system for EGFP labeling of the shot-hole pathogen Wilsonomyces carpophilus

**DOI:** 10.3389/fmicb.2026.1917079

**Published:** 2026-09-24

**Authors:** Xinmei Zhou, Chuli Liu, Jiayuan Ouyang, Yiheng Zhang, Dianguang Xiong, Chengming Tian, Yusufjon Gafforov, Rong Ma

**Affiliations:** 1 College of Forestry and Landscape Architecture, Xinjiang Agricultural University, Urumqi, China; 2 College of Forestry, Beijing Forestry University, Beijing, China; 3 Department of Botany and Genetics, National University of Uzbekistan, Tashkent, Uzbekistan; 4 Department of Medicine, Faculty of Medicine, Alfraganus University, Tashkent, Uzbekistan

**Keywords:** EGFP reporter, enzymatic cell wall digestion, filamentous ascomycete, fungal genetic manipulation, host–pathogen interaction, hygromycin B resistance, osmotic stabilization, stone fruit disease

## Abstract

*Wilsonomyces carpophilus* is a fungal pathogen causing shot-hole disease in stone fruit trees, including wild apricot populations in the Tianshan wild fruit forests. The absence of an effective genetic transformation system has limited molecular studies of this pathogen. In this study, we established a PEG-mediated protoplast transformation system for *W. carpophilus* strain G052 5 m2. Key factors affecting protoplast preparation, including mycelial culture duration, enzyme combination, digestion time, and osmotic stabilizer, were evaluated. The optimal conditions consisted of 24-h-old mycelia, enzymatic digestion with 20 g/L Driselase and 25 g/L Snailase for 5 h, and 1.2 mol/L NaCl as the osmotic stabilizer, yielding up to 4.13 × 10^6^ protoplasts/mL. The PEG-mediated transformation efficiency was calculated as 1.75 transformants per microgram of plasmid DNA under the optimized conditions. Both PCR detection and EGFP fluorescence observation successfully validated the exogenous gene expression in fungal transformants. No obvious differences in colony morphology, vegetative growth, and pathogenicity were observed between transformants and the wild-type strain. This study provides the first PEG-mediated protoplast transformation system for *W. carpophilus* and establishes a practical molecular tool for future studies on gene function, pathogenicity mechanisms, and host–pathogen interactions in this important stone fruit pathogen.

## 1 Introduction

The Tianshan wild fruit forests constitute an important natural reservoir of temperate fruit-tree diversity and a key germplasm center for the wild relatives of cultivated fruit crops. *Prunus armeniaca*, a dominant relict species in this ecosystem, represents an ancestral resource of cultivated apricot and preserves valuable genetic information for temperate deciduous fruit trees of the Northern Hemisphere (He et al., 2007; Xu et al., 2019; Xiayida et al., 2024). Conservation of wild apricot populations carries substantial scientific and practical significance (Liu, 2010).

*Wilsonomyces carpophilus* is an ascomycetous fungal pathogen associated with shot-hole disease of stone fruit trees. The pathogen is widely distributed globally and has also been reported from several regions of China (Dai, 1979; Ahmadpour et al., 2009; Marin-Felix et al., 2017). It infects economically important hosts such as *P. armeniaca* and *P. persica*, causing symptoms that include leaf perforation, brown lesions on fruit, and shoot dieback (Adaskaveg et al., 1990; Ivanová et al., 2012; Grantina-Ievina and Stanke, 2015; Narmani et al., 2018; Ye et al., 2020). Severe disease outbreaks can lead to substantial yield losses in stone fruit production, with reported losses ranging from 30 to 90% in susceptible hosts (Bubici et al., 2010; Narmani et al., 2018). Field incidence rates above 75% have also been recorded in several regions, including Mexico and India (Bubici et al., 2010; Tovar-Pedraza et al., 2013). In the Tianshan wild fruit forests, *W. carpophilus* was confirmed to infect wild apricot in 2019 (Cheng et al., 2019). Subsequent surveys showed a high incidence of diseased wild apricot trees, with an incidence rate of 92.94% and a disease severity index of 48.35 (Shi et al., 2025). These findings indicate that shot-hole disease poses a serious threat to the persistence of wild apricot populations and may increase the risk of cross-infection among related stone fruit hosts.

Current management of *W. carpophilus* relies mainly on chemical control, together with agricultural and integrated field management practices (Gravite and Kaufmane, 2013; Mitre et al., 2015; García-Galavís et al., 2009; Zhao et al., 2000; Karlidag et al., 2010; Azmy and Korra, 2010; Nabi et al., 2018). However, the Tianshan wild fruit forests are ecologically sensitive, and long-term or large-scale pesticide use may increase the risk of fungicide resistance and disturb soil microbial communities. Such effects are inconsistent with the conservation goals for wild fruit forest ecosystems and highlight the need for a better understanding of the pathogen’s biology, virulence mechanisms, and interaction with its host (Chen et al., 2023). Nevertheless, molecular research on *W. carpophilus* is still severely limited, largely due to the lack of mature and stable genetic transformation systems for this pathogen. An *Agrobacterium tumefaciens*-mediated transformation (ATMT) system has been reported for *W. carpophilus* (Rasool et al., 2020); however, no PEG-mediated protoplast transformation protocol is currently available for this pathogen. Compared with ATMT, PEG-mediated protoplast transformation requires less experimental time and no specialized binary vectors, providing an alternative complementary tool for gene functional analysis.

Genetic transformation is an essential approach for functional studies of filamentous fungi. Several transformation methods have been used in fungal research, including PEG-mediated protoplast transformation, electroporation, restriction enzyme-mediated transformation, and *Agrobacterium*-mediated transformation (Woloshuk et al., 1989; Michielse et al., 2005; Han et al., 2018). Among these, PEG-mediated protoplast transformation is widely used because it is relatively simple, cost-effective, and does not require specialized equipment (Ferrer et al., 1985; Díaz et al., 2019; Ullah et al., 2020; Liang et al., 2021; Zhang et al., 2022). This method depends on the preparation of viable protoplasts by enzymatic removal of the fungal cell wall, followed by PEG- and CaCl2-assisted uptake of exogenous DNA and selection of stable transformants (Hinnen et al., 1978; Case et al., 1979; Ballance et al., 1983; Martín, 2015; Shen et al., 2017). Because the fungal cell wall is mainly composed of glucans and chitin, its composition and rigidity can strongly affect the efficiency of protoplast release and DNA uptake (Geoghegan et al., 2017; Kemppainen et al., 2020). Therefore, the optimization of mycelial age, enzyme composition, digestion time, and osmotic stabilizer is a critical step in establishing an effective transformation system (Dobrowolska and Staczek, 2009; Agrawal et al., 2015; Aloulou-Abdelkefi et al., 2017; Adeleye et al., 2019; Chu et al., 2014).

Although PEG-mediated transformation protocols have been developed for several filamentous fungi, no single protocol applies to all species because fungal cell wall composition and physiological responses differ among taxa and strains. A PEG-mediated transformation system has been reported for *Trichothecium roseum*, an ascomycetous fungus, using enzymatically prepared protoplasts and *egfp*-based transformant screening (Dai et al., 2019). However, preliminary attempts to adapt this system to *W. carpophilus* yielded low protoplast yields and failed transformations, indicating that a species-specific protocol is required. To date, no efficient genetic transformation system has been reported for *W. carpophilus*, thereby limiting molecular studies of gene function and pathogenicity in this pathogen.

In this study, we established and optimized a PEG-mediated protoplast transformation system for *W. carpophilus*. The effects of mycelial culture duration, enzyme combinations, digestion conditions, and osmotic stabilizers on protoplast production were evaluated. The transformation system was then validated using the PCT74-EGFP plasmid as a reporter vector. This work provides a practical genetic tool for future studies on virulence-related genes, pathogenicity mechanisms, and host–pathogen interactions in *W. carpophilus*.

## 2 Materials and methods

### 2.1 Fungal strain, plasmid, and major enzymes

The *Wilsonomyces carpophilus* strain G052 5 m2 used in this study was isolated from diseased wild apricot fruit collected in Huocheng County, Ili Kazakh Autonomous Prefecture, Xinjiang, China, in 2018. The strain was identified by our laboratory and deposited in the China Forestry Culture Collection Center under accession number CFCC 71543. Cultures were maintained on potato dextrose agar (PDA) medium at 4°C and transferred to fresh medium before use.

The recombinant plasmid PCT74-EGFP was used as the transformation vector. This plasmid carries the enhanced green fluorescent protein gene (*egfp*) as a reporter marker and the hygromycin B phosphotransferase gene (*hyg*) as a selectable marker. The *egfp* expression cassette is driven by the ToxA promoter from *Pyrenophora tritici-repentis*, with the NOS terminator serving as the transcription terminator. The plasmid was purchased from Hunan Fenghui Biotechnology Co., Ltd., China.

Three cell wall-degrading enzymes were applied for protoplast isolation of *W. carpophilus*. Driselase (Cat. No. D9515) and Lysing Enzymes (Cat. No. R1020) were purchased from Sigma-Aldrich Company. Snailase (Cat. No. S8280) was obtained from Solarbio Science and Technology Co., Ltd., Beijing, China. All enzyme powders were stored at −20°C before use and prepared into working solutions immediately prior to protoplast digestion. The Lysing Enzymes working solution was freshly prepared and filter-sterilized immediately prior to protoplast isolation. The Protoplast Transformation Component (PTC) solution was also freshly prepared, filter-sterilized and used on the same day. Detailed information on reagents and culture media is shown in Table 1.

**TABLE 1:** Medium composition for *Wilsonomyces carpophilus* genetic transformation.

Medium	Composition
Potato dextrose agar (PDA) medium	Potato 200 g/L, glucose 20 g/L, agar 15 g/L
Yeast extract peptone dextrose (YEPD)	Yeast Extract 3 g/L, bacterial peptone 10 g/L, glucose 20 g/L
Regeneration solid medium (TB_3_ solid medium)	Yeast Extract 3 g/L, casamino acids 3 g/L, sucrose 200 g/L, agar 7 g/L
Regeneration liquid medium (TB_3_ liquid medium)	Yeast Extract 3 g/L, casamino acids 3 g/L, sucrose 200 g/L
Antibiotic medium	Potato 200 g/L, glucose 20 g/L, agar 15 g/L, 25 μg/mL hygromycin B
Sucrose-Tris-CaCl_2_ (STC) buffer	Sucrose 200 g/L, 1 M Tris-HCl (pH 8.0) 50 mL/L, CaCl_2_ 5.549 g/L
Protoplast transformation component (PTC) solution	PEG4000 400 g/L, solvent: STC buffer

### 2.2 Hygromycin B sensitivity assay

To determine the appropriate concentration of hygromycin B for selecting transformants, the sensitivity of *W. carpophilus* strain G052 5 m2 to hygromycin B was tested. The strain was grown on PDA plates at 25°C in the dark for 5 days. Mycelial plugs, 5 mm in diameter, were taken from the actively growing colony margin and transferred onto PDA plates supplemented with hygromycin B at concentrations of 0, 5, 10, 15, 20, 25, 30, and 35 μg/mL. PDA medium without hygromycin B was used as the control. Each treatment was performed with five replicates. Plates were incubated at 25°C in the dark, and colony growth was observed and recorded after 15 days.

### 2.3 Preparation and purification of protoplasts

#### 2.3.1 Mycelial culture

The *W. carpophilus* strain stored at 4°C was transferred to fresh PDA plates and incubated at 25°C for 7 days. Subsequently, it was sub-cultured onto new PDA plates and incubated for another 3 days. Three fresh 5-mm-diameter mycelial plugs excised from the actively growing colony margin were inoculated into 250-mL Erlenmeyer flasks containing 100 mL of yeast extract-peptone-dextrose (YEPD) liquid medium. Cultures were incubated at 25°C and 150 rpm on a rotary shaker. Mycelia were collected by filtration through sterile Miracloth with a pore size of 22 μm and washed three to four times with sterile osmotic buffer before enzymatic digestion. For protoplast enzymatic digestion, the washed mycelia were resuspended in sterile enzyme solution at a ratio of 20 mL enzyme solution per gram of wet mycelium.

#### 2.3.2 Enzymatic digestion of mycelia

Washed mycelia were resuspended in 20 mL of sterile enzyme solution and incubated at 30°C and 70 rpm with gentle shaking. Samples were taken at regular intervals and examined under a microscope to monitor protoplast release. The effects of enzyme composition, mycelial culture duration, digestion time, and osmotic stabilizer were evaluated as described below (Table 2).

**TABLE 2:** Summary of optimized conditions for protoplast preparation and PEG-mediated transformation of *Wilsonomyces carpophilus* strain G052 5 m2.

Parameter	Tested conditions	Selected condition	Main result
Selection antibiotic	Hygromycin B gradient	10 μg/mL hygromycin B	Inhibited growth of the wild-type strain
Enzyme combination	25 g/L Snailase; 25 g/L Snailase + 25 g/L Lysing Enzymes; 20 g/L Driselase + 25 g/L Snailase; 25 g/L Snailase + 20 g/L Driselase + 25 g/L Lysing Enzymes	20 g/L Driselase + 25 g/L Snailase	1.08 × 10^6^ protoplasts/mL, (tested with 1.2 mol/L KCl, 42-h mycelia)
Mycelial culture duration	8, 16, 24, and 42 h	24 h	1.67 × 10^6^ protoplasts/mL (under 1.2 mol/L KCl, 20 g/L Driselase, and 25 g/L Snailase)
Digestion time	Hourly monitoring during enzymatic digestion	5 h	Selected based on yield and experimental efficiency
Osmotic stabilizer	0.7 mol/L KCl, 1.2 mol/L KCl, 0.7 mol/L NaCl, 1.2 mol/L NaCl	1.2 mol/L NaCl	4.13 × 10^6^ protoplasts/mL (under 20 g/L Driselase and 25 g/L Snailase, 24-h mycelia, 5 h digestion,
Transformation vector	PCT74-EGFP	PCT74-EGFP, 4μg	*hyg* and *egfp* markers used
Transformant verification	Antibiotic selection, PCR, EGFP fluorescence	Transformation confirmed by PCR and fluorescence microscopy	Seven PCR-positive transformants

#### 2.3.3 Collection and washing of protoplasts

After enzymatic digestion, the hydrolysate was filtered through two layers of sterile Miracloth to remove undigested mycelial fragments. The filtrate containing protoplasts was collected in 50-mL centrifuge tubes and centrifuged at 4°C and 3,000 rpm for 10 min. The supernatant was discarded, and the protoplast pellet was gently resuspended in Sucrose-Tris-CaCl_2_ (STC) buffer. The protoplast concentration was adjusted to approximately 1 × 10^8^ protoplasts/mL for subsequent transformation experiments. A hemocytometer was used to count protoplasts. The formula for cell density is shown below:


$$C=\frac{N}{80}\times 400\times 10,000$$

In the formula:

C: Protoplast concentration, protoplasts/mL;

N: Total number of protoplasts counted in five medium squares;

(80 = 5 × 16), representing the total number of small squares included in the five medium squares;

400: Total number of small squares in the central large square of the hemocytometer;

10,000: Volume conversion factor.

### 2.4 PEG-mediated protoplast transformation

For PEG-mediated transformation, 250 μL of freshly prepared protoplast suspension (adjusted to 1 × 10^8^ protoplasts/mL, see section 2.3.3) was transferred into a sterile 50-mL centrifuge tube. Four micrograms of PCT74-EGFP plasmid DNA were added to the protoplast suspension, mixed gently, and incubated at room temperature for 20 min. Then, 1.2 mL of 40% PTC solution was added, mixed carefully, and incubated for another 20 min at room temperature. Subsequently, 3 mL of TB_3_ liquid medium was added and mixed gently, followed by the addition of another 5 mL of TB_3_ liquid medium. The mixture was incubated at 25°C and 70 rpm for 14–20 h to allow protoplast regeneration.

After regeneration, the culture was mixed with TB_3_ solid medium containing hygromycin B and poured into plates. Plates were incubated at 25°C in the dark until resistant colonies appeared. Putative transformants were transferred to fresh PDA plates containing hygromycin B for further screening.

### 2.5 Optimization of protoplast preparation conditions

#### 2.5.1 Effect of mycelial culture duration

To determine the optimal mycelial culture duration for protoplast preparation, mycelia cultured in YEPD liquid medium for 8, 16, 24, and 42 h were collected and subjected to enzymatic digestion. The enzyme system consisted of 20 g/L Driselase and 25 g/L Snailase in 1.2 mol/L KCl buffer. Digestion was performed at 30°C and 70 rpm. Samples were collected every hour, and protoplast concentration was determined using a hemocytometer.

#### 2.5.2 Effect of enzyme combinations

To compare the effects of different enzyme combinations on protoplast yield, four enzyme treatments were tested under 1.2 mol/L KCl osmotic stabilization: 25 g/L Snailase; 25 g/L Snailase + 25 g/L Lysing Enzymes; 20 g/L Driselase + 25 g/L Snailase; and 25 g/L Snailase + 20 g/L Driselase + 25 g/L Lysing Enzymes. Mycelia were incubated with the enzyme solutions at 30°C and 70 rpm. Protoplast yield was measured at regular intervals to determine the most effective enzyme combination.

#### 2.5.3 Effect of osmotic stabilizers

To determine the optimal osmotic stabilizer, mycelia were digested using the selected enzyme combination of 20 g/L Driselase and 25 g/L Snailase for 5 h. Four osmotic stabilizers were tested: 0.7 mol/L KCl, 1.2 mol/L KCl, 0.7 mol/L NaCl, and 1.2 mol/L NaCl. Protoplast yield was measured under the same digestion conditions, and the stabilizer producing the highest protoplast yield was selected for subsequent transformation experiments.

### 2.6 Screening and molecular verification of transformants

#### 2.6.1 PCR verification

Putative transformants showing growth on hygromycin B-containing medium were transferred to fresh PDA plates containing hygromycin B and screened for three successive rounds. Fresh mycelia were collected from resistant colonies, and genomic DNA was extracted using the CTAB method. PCR was performed using primers specific to the PCT74-EGFP plasmid:M13-7F, 5′-GGC GGT GCT ACA GAG TTC TT-3′, and M13-7R, 5′-GCG TCA GAC CCC GTA GAA AA-3′. The expected amplification product was approximately 236 bp.

PCR was performed in a 25-μL reaction volume under the following conditions: initial denaturation at 95°C for 5 min; 30 cycles of denaturation at 95°C for 30 s, annealing at 59°C for 30 s, and extension at 72°C for 30 s; followed by a final extension at 72°C for 10 min. Genomic DNA from the wild-type strain was used as the negative control, PCT74-EGFP plasmid DNA was used as the positive control, and sterile water was used as the blank control. PCR products were separated by electrophoresis on a 2.0% agarose gel.

#### 2.6.2 Fluorescence microscopy

To verify EGFP expression in the transformants, fresh hyphae from PCR-positive colonies were mounted on glass slides. EGFP signals of the hyphae of transformants were observed using a fluorescence microscope (ECLIPSE Ni-E, Nikon, Tokyo, Japan). The FITC filter set (excitation 465–495 nm, dichroic mirror DM 505, emission 512–550 nm) was used for GFP signal acquisition. Digital images were captured with NIS-Elements D software. The wild-type strain was used as the negative control.

For mitotic stability verification, transformants were consecutively sub-cultured for five passages on non-selective medium without hygromycin B. After five passages, strains were transferred back to hygromycin B-containing selective plates for resistance detection, and EGFP fluorescence was re-examined simultaneously.

### 2.7 Phenotypic characterization of fluorescent strains

Wild-type and transformant strains were activated on potato dextrose agar (PDA) plates. Mycelial plugs of each strain were inoculated onto fresh PDA plates with three biological replicates. All plates were incubated at 28 °C for 20 days. Colony area was quantified using ImageJ-Fiji software. Mycelial growth rate was calculated as the average radial colony growth divided by incubation time.

### 2.8 Pathogenicity assay

For in-vivo inoculation, wild apricot leaves with consistent growth status, size and health condition were selected and surface-sterilized with 75% ethanol. Subsequently, the leaves were thoroughly rinsed with sterile distilled water and air-dried. Each leaf was wounded at symmetrical positions relative to the veins on the adaxial surface using a No. 2 insect pin (0.38 mm diameter). Activated wild-type strain and transformants were inoculated onto the wound sites, while sterile PDA was applied for the control group (CK). Following inoculation, leaves were kept moist using absorbent cotton saturated with sterile distilled water. Disease development was monitored over time to evaluate pathogenicity.

### 2.9 Statistical analysis

Data were organized using Microsoft Excel and analyzed using GraphPad Prism 10.1.2. Protoplast yield data are presented as mean ± standard error. Two-way analysis of variance was used to evaluate the effects of enzyme combination and digestion time on protoplast yield. One-way analysis of variance followed by Tukey’s multiple comparison test was used to compare the effects of different osmotic stabilizers. Differences were considered statistically significant at *p* ≤ 0.05.

## 3 Results

### 3.1 Hygromycin B sensitivity of *Wilsonomyces carpophilus* strain G052 5 m2

To establish a reliable selection system for PEG-mediated transformation, the sensitivity of *W. carpophilus* strain G052 5 m2 to hygromycin B was first evaluated. The wild-type strain was cultured on PDA medium supplemented with a gradient of hygromycin B concentrations, and colony growth was compared with that on antibiotic-free PDA medium. The strain grew normally on the control medium, indicating that the culture conditions supported active mycelial development in the absence of selection pressure. In contrast, the addition of hygromycin B progressively inhibited mycelial growth as the antibiotic concentration increased.

Growth inhibition was evident at 10 μg/mL hygromycin B, and this concentration was sufficient to suppress the growth of the recipient strain under the tested conditions (Figure 1). In subsequent transformation experiments, 25 mg/L hygromycin B was applied during primary screening to reduce the occurrence of false-positive colonies (Sun et al., 2009; Liu et al., 2017).

**FIGURE 1:**
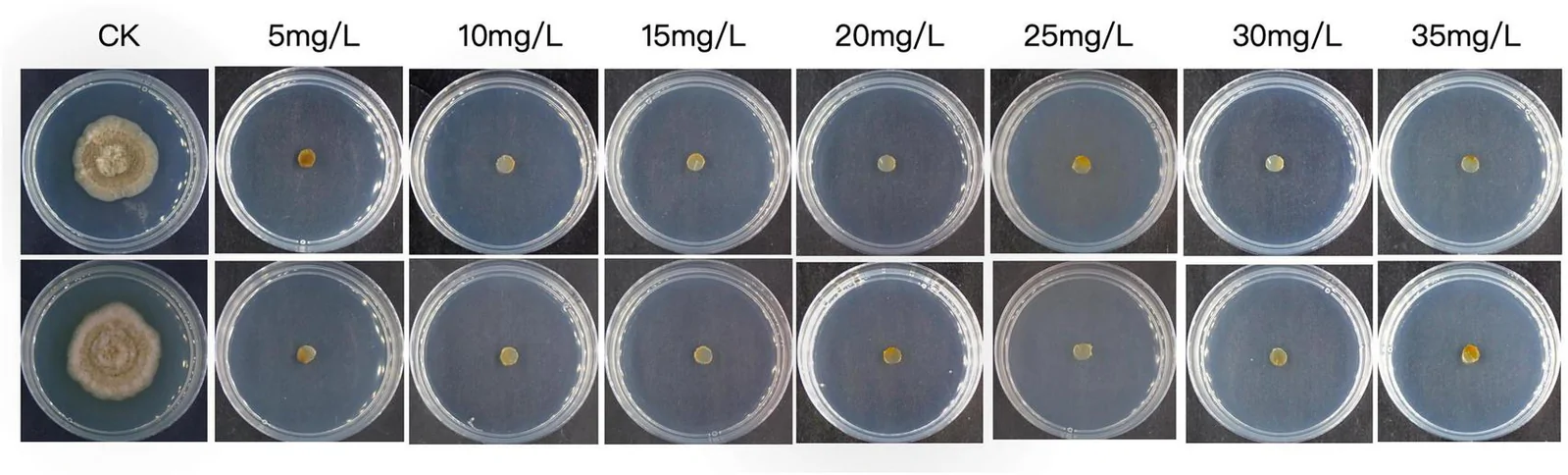
Sensitivity of *Wilsonomyces carpophilus* strain G052 5 m2 to different concentrations of hygromycin B.

### 3.2 Effect of enzyme combinations on protoplast release

The release of protoplasts from *W. carpophilus* mycelia was strongly affected by the composition of the cell wall-degrading enzyme mixture. Under osmotic stabilization with 1.2 mol/L KCl, four enzyme treatments were compared to determine their effects on protoplast production. Snailase alone produced the lowest protoplast yield, indicating that this enzyme was insufficient for efficient digestion of the *W. carpophilus* cell wall under the tested conditions. The combination of Snailase and Lysing Enzymes increased protoplast release to some extent, but the yield remained lower than that obtained with the Driselase-containing treatment.

Among the tested enzyme combinations, 20 g/L Driselase combined with 25 g/L Snailase produced the highest protoplast yield, reaching 1.08 × 10^6^ protoplasts/mL (Figure 2). However, the addition of Lysing Enzymes to this combination did not further improve protoplast release. Instead, the yield decreased when 25 g/L Lysing Enzymes was included together with Driselase and Snailase. These results show that the two-enzyme combination of 20 g/L Driselase and 25 g/L Snailase was the most effective treatment among those tested and was therefore selected for further optimization.

**FIGURE 2:**
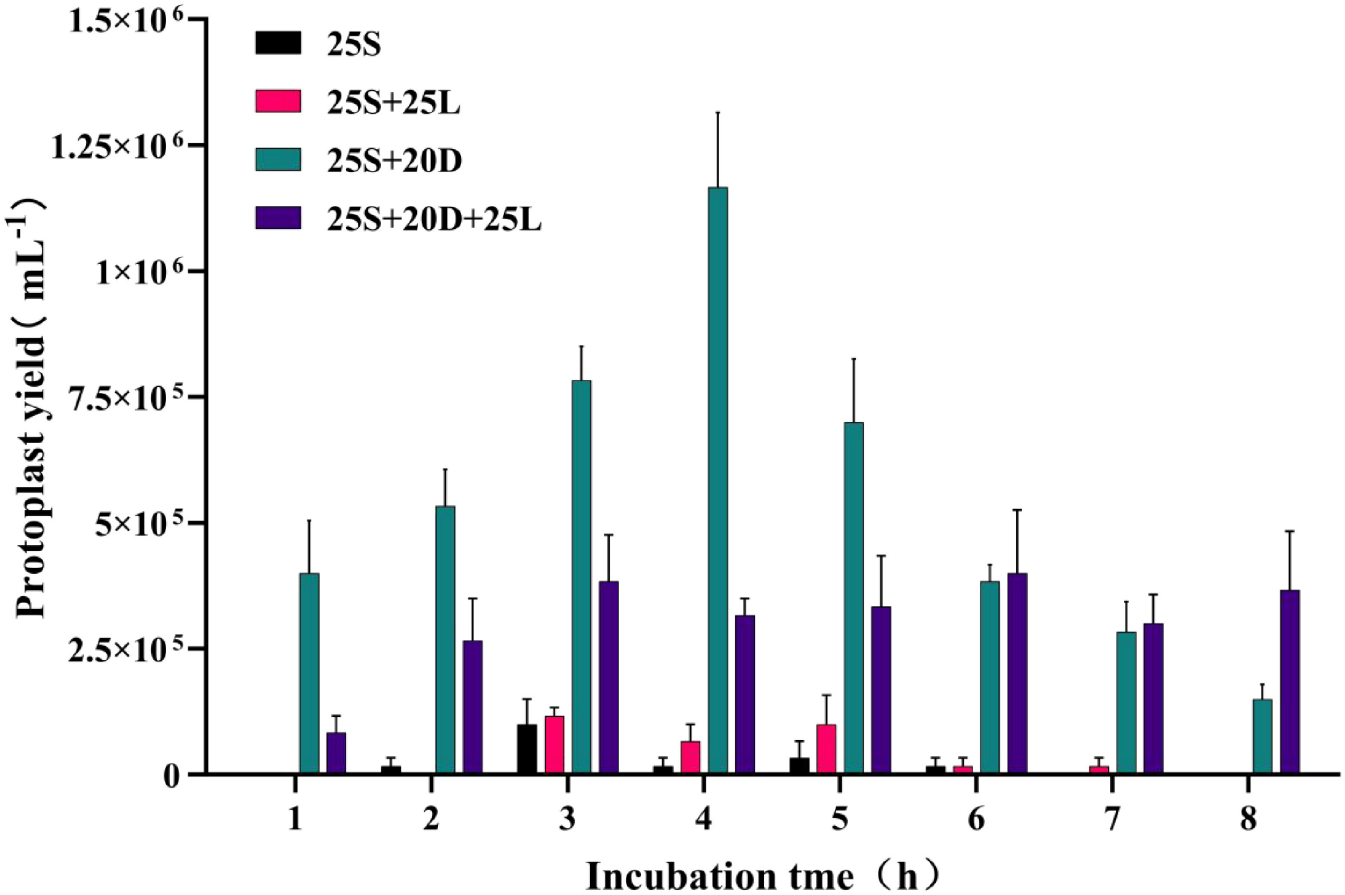
Effect of different enzyme combinations on protoplast release from *Wilsonomyces carpophilus* strain G052 5 m2 mycelia.

### 3.3 Effect of mycelial culture duration and digestion time on protoplast yield

The physiological state of the mycelium is an important factor affecting protoplast preparation. To determine the optimal mycelial culture duration, mycelia cultured for 8, 16, 24, and 42 h were subjected to enzymatic digestion, For all tested groups, 1 g of washed wet mycelium was resuspended in 20 mL enzyme solution following the standardized biomass-enzyme ratio described in section 2.3.2, and protoplast yield was monitored over time. Mycelia cultured for 8 h produced the lowest protoplast yield, suggesting that the biomass at this stage was insufficient for efficient protoplast release. As the culture duration increased, protoplast production improved, indicating that young but sufficiently developed mycelia were more suitable for enzymatic digestion.

The 24-h-old mycelia showed the best overall performance among the tested culture durations. After 5 h of enzymatic digestion, the protoplast yield reached 1.67 × 10^6^ protoplasts/mL. Continuous monitoring showed that the yield further increased to 2.32 × 10^6^ protoplasts/mL after 7 h of digestion (Figure 3). However, extending the mycelial culture duration to 42 h reduced protoplast production, with a yield of 1.30 × 10^6^ protoplasts/mL. This decline indicates that older mycelia were less suitable for enzymatic hydrolysis under the tested conditions. Considering both protoplast yield and experimental efficiency, 24-h-old mycelia and 5 h of enzymatic digestion were selected for subsequent experiments.

**FIGURE 3:**
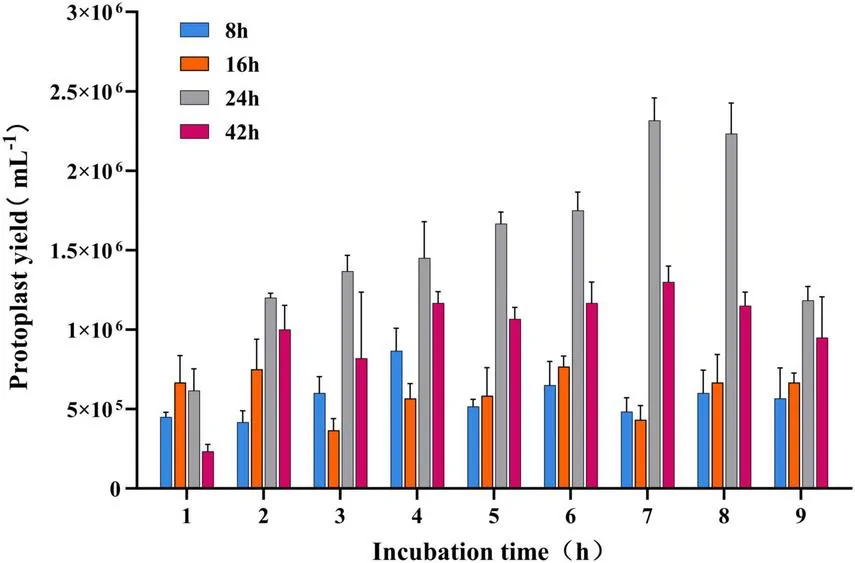
Effect of mycelial culture duration and enzymatic digestion time on protoplast yield in *Wilsonomyces carpophilus* strain G052 5 m2.

### 3.4 Effect of osmotic stabilizers on protoplast yield

The osmotic stabilizer used during enzymatic digestion also affected protoplast production. To identify the most suitable osmotic condition, four stabilizers were tested using the selected enzyme combination of 20 g/L Driselase and 25 g/L Snailase: 0.7 mol/L KCl, 1.2 mol/L KCl, 0.7 mol/L NaCl, and 1.2 mol/L NaCl. All four stabilizers supported protoplast release, but the yields differed among treatments, indicating that both the type and concentration of the osmotic stabilizer influenced the efficiency of protoplast preparation.

The highest protoplast yield was obtained with 1.2 mol/L NaCl, reaching 4.13 × 10^6^ protoplasts/mL (Figure 4). This yield was higher than those obtained with the other tested osmotic stabilizers. In contrast, 1.2 mol/L KCl produced a lower yield of 1.95 × 10^6^ protoplasts/mL under the same enzymatic conditions. Based on these results, 1.2 mol/L NaCl was selected as the optimal osmotic stabilizer for protoplast preparation of *W. carpophilus* strain G052 5 m2.

**FIGURE 4:**
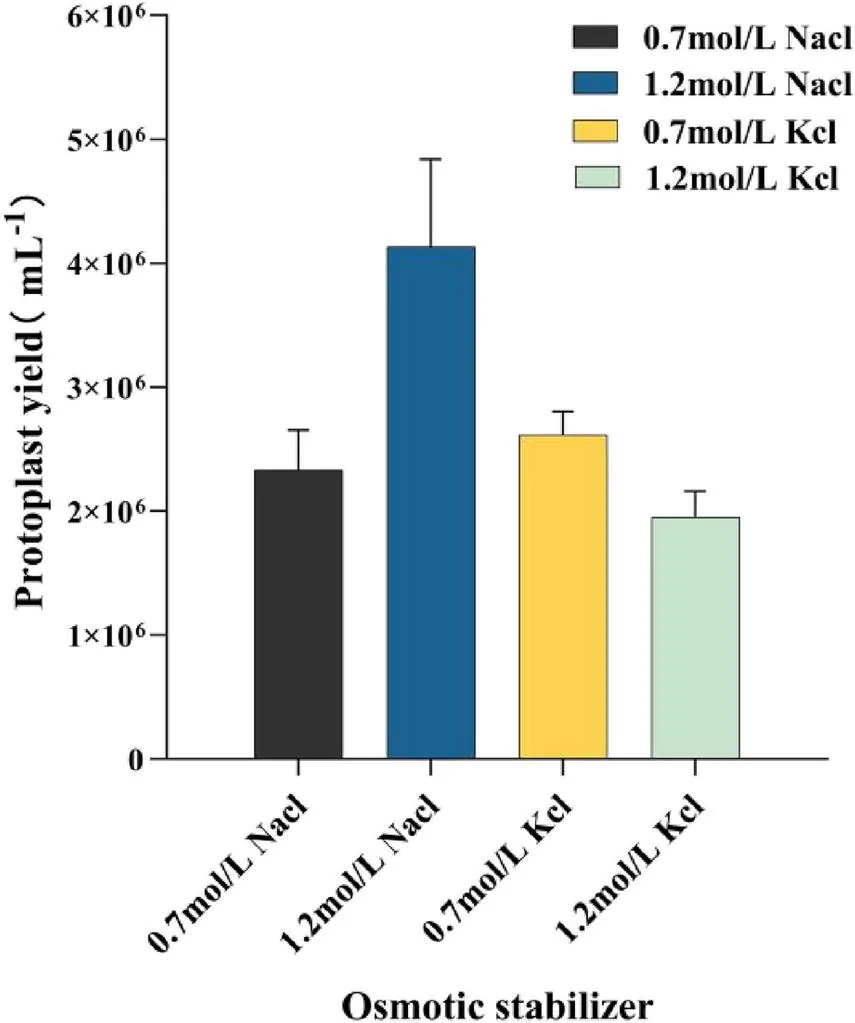
Effect of different osmotic stabilizers on protoplast yield from *Wilsonomyces carpophilus* strain G052 5 m2 mycelia.

### 3.5 Microscopic observation of protoplast release and morphological characteristics

Microscopic observation revealed that protoplasts of *W. carpophilus* were mainly released from hyphal apices (Figures 5B–D), whereas lateral protoplast release from hyphae was also observed (Figure 5E). The diameter of the obtained protoplasts ranged from 5.02 to 14.58 μm, with an average diameter of 8.57 μm. Representative microscopic fields of digested mycelia and purified protoplasts are shown in Figure 5A.

**FIGURE 5:**
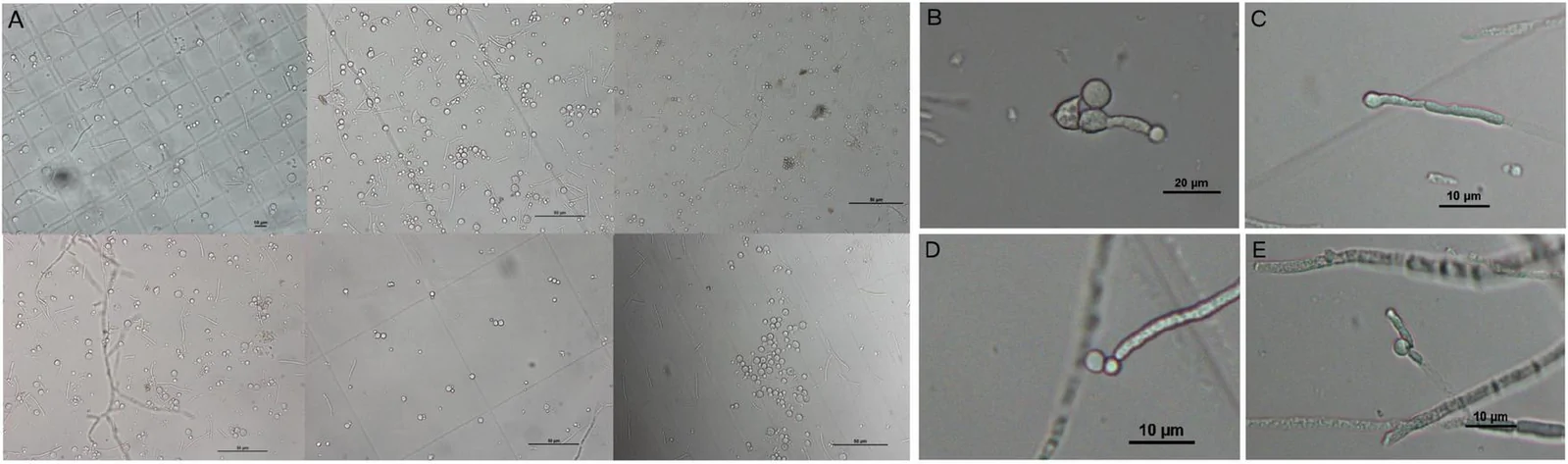
Protoplast formation and release of *Wilsonomyces carpophilus*. **(A)** Morphology of enzymatically digested protoplasts. **(B–D)** Apical release of protoplasts from hyphae. **(E)** Lateral release of protoplasts from hyphae.

### 3.6 PEG-mediated transformation and verification of transformants

The optimized protoplast preparation conditions were used to establish PEG-mediated transformation of *W. carpophilus*. Freshly prepared protoplasts were transformed with the PCT74-EGFP plasmid, which carries the *hyg* selectable marker and the *egfp* reporter gene. After antibiotic selection, eighteen resistant colonies were obtained from one transformation experiment. For three successive rounds of purification, small mycelial fragments were excised from the growing margin of each resistant colony and transferred onto fresh selective medium containing the same concentration of hygromycin B used for primary transformant screening, to eliminate unstable heterokaryons and false-positive transformants. Following three rounds of subculture, a total of nine stably hygromycin B-resistant transformant colonies were obtained. Fresh mycelia were harvested from these stably purified cultures for subsequent PCR detection and fluorescence observation. PCR amplification was then performed using primers specific to the PCT74-EGFP plasmid. The expected 236-bp fragment was detected in seven transformants, with a transformation positive rate of 77.78%, whereas no amplification was observed in the wild-type strain or blank control (Figure 6). The presence of the expected PCR product in the selected colonies confirmed that the introduced plasmid sequence was present in the transformants.

**FIGURE 6:**
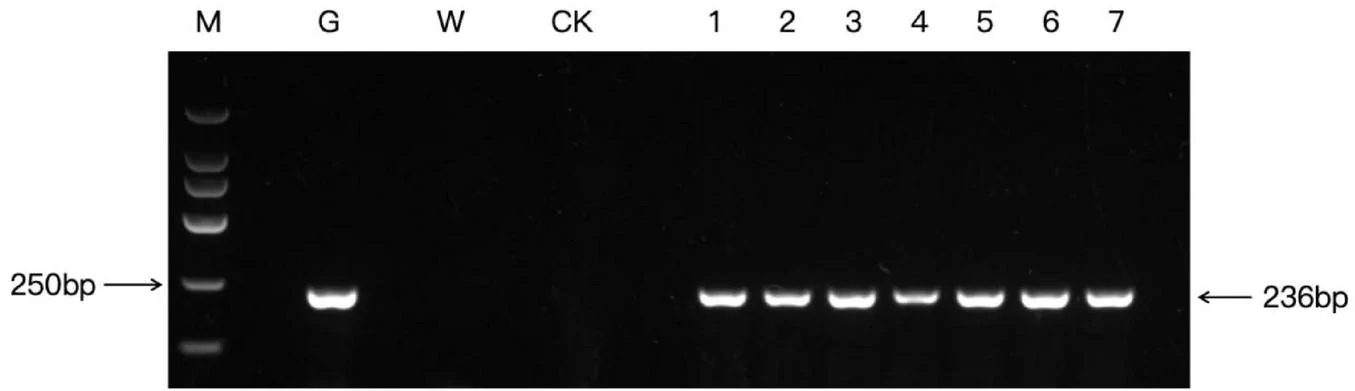
PCR verification of *Wilsonomyces carpophilus* transformants generated using the PCT74-EGFP plasmid. M, DL2000 DNA marker; G, PCT74-EGFP plasmid; W, wild-type strain; CK, blank control; lanes 1–7, EGFP transformants G1–G7.

Fluorescence microscopy was used to further verify reporter gene expression. All transformants were derived from a transformation experiment with identical genetic backgrounds. Two out of the seven PCR-positive transformants were randomly selected for fluorescence microscopic observation. Compared with the wild-type control, EGFP-tagged transformants showed green fluorescence in the hyphae (Figure 7), indicating expression of the *egfp* reporter gene. The combined results of antibiotic selection, PCR amplification, and EGFP fluorescence observation confirmed that the PCT74-EGFP plasmid was successfully introduced into *W. carpophilus* protoplasts using the optimized PEG-mediated transformation protocol.

**FIGURE 7:**
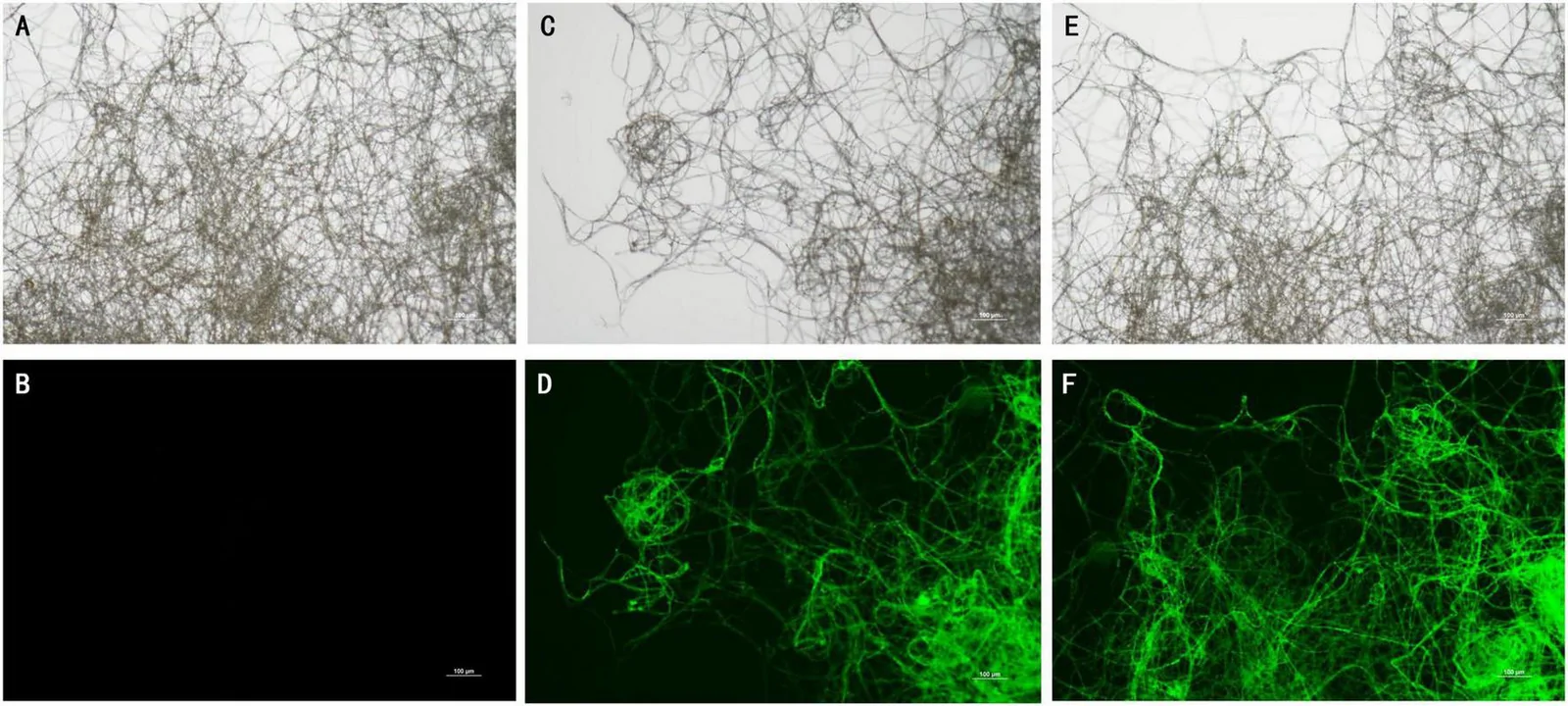
Fluorescence microscopy observation of wild-type and EGFP-tagged *Wilsonomyces carpophilus* strain G052 5 m2. **(A,B)** Wild-type hyphae observed under bright-field and fluorescence channels, respectively; **(C–F)** EGFP-tagged transformants observed under bright-field and fluorescence channels. Green fluorescence in the transformed hyphae indicates expression of the *egfp* reporter gene. Scale bars = 100 μm.

Transformation efficiency was calculated based on the number of genuine transformants that survived three successive rounds of hygromycin-B purification and yielded target amplicons in PCR assays. When 4 μg of plasmid DNA was added to 250 μL protoplast suspension at a concentration of 1 × 10^8^ protoplasts/mL, seven PCR-positive transformants were obtained, with a transformation efficiency of 1.75 transformants per microgram of plasmid DNA.

### 3.7 Analysis of phenotypic differences between wild-type strains and transformants

Phenotypic analysis was carried out using pre-validated positive transformants to eliminate potential interference from false-positive strains. No significant differences were detected in colony morphology, average colony area, and mycelial growth rate between the wild-type (WT) strain and the transformant (*p* > 0.05, marked as “ns”) (Figures 8A,B). Consistent with these quantitative results, visual observation of colony phenotypes on PDA medium also revealed no obvious morphological variations between WT and transformant (Figure 9). Collectively, these results indicated that genetic transformation did not affect the vegetative growth of the strain under the tested culture conditions.

**FIGURE 8:**
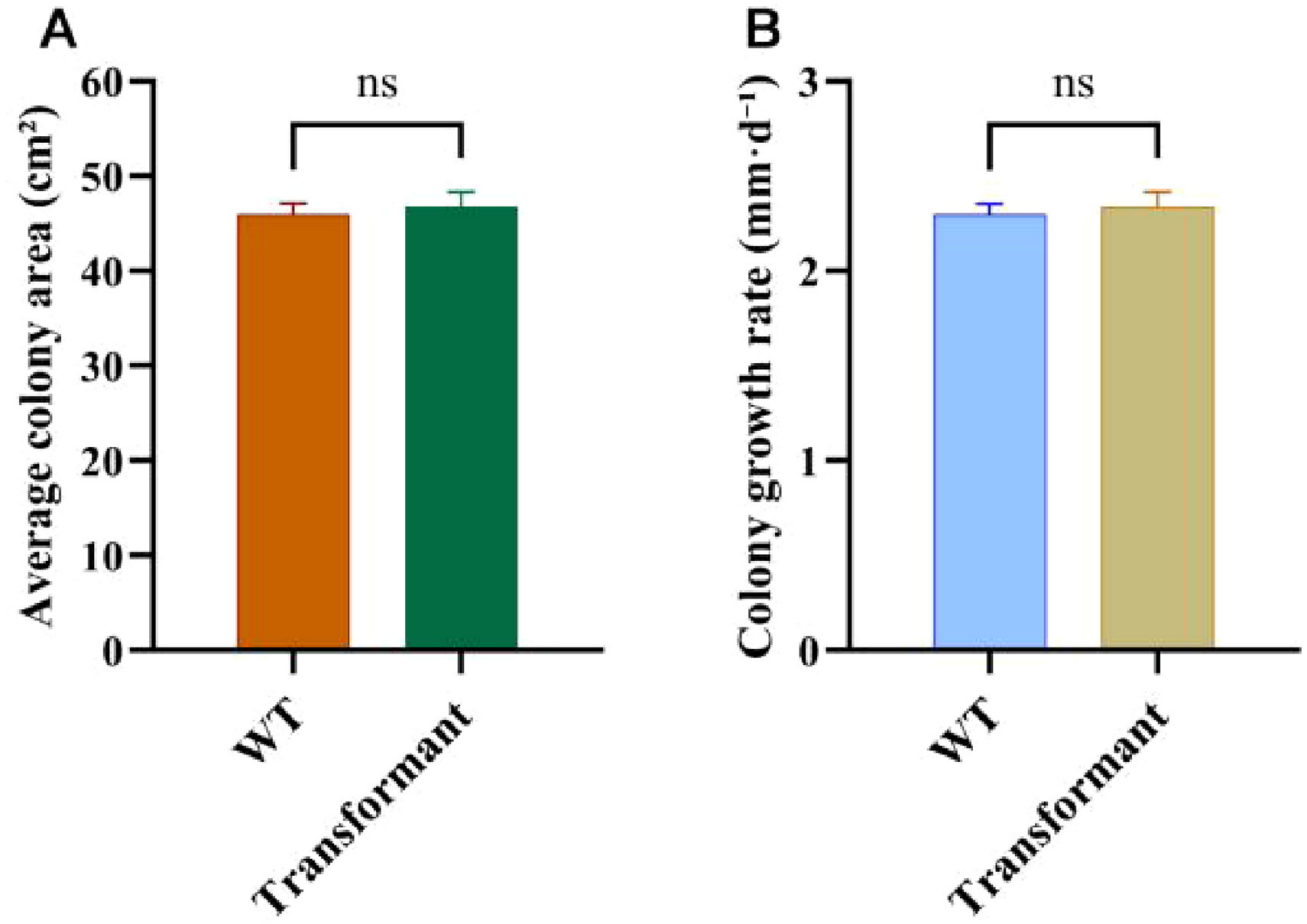
Comparison of the average colony area and colony growth rate between wild-type (WT) strain and transformant strain. **(A)** Average colony area. **(B)** Colony growth rate. WT represents the wild-type strain, and Transformant represents the genetically transformed strain. ns indicates no significant difference between the two groups (*p* > 0.05).

**FIGURE 9:**
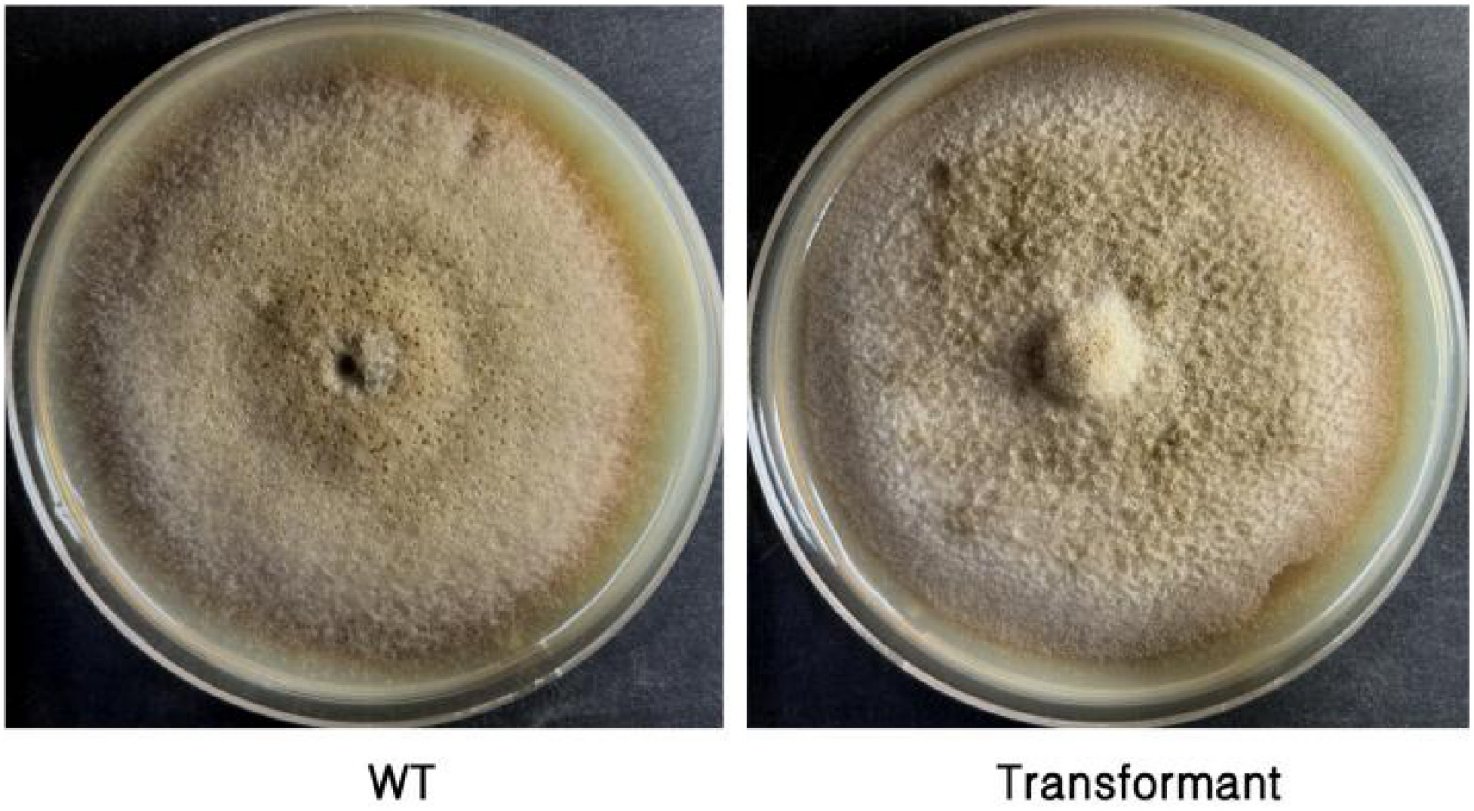
Colonial phenotypes of wild-type (WT) strain and transformant strain cultured on solid medium.

### 3.8 Mitotic stability of EGFP-expressing transformants

The mitotic stability of EGFP-expressing transformants was evaluated by five successive rounds of subculture on solid non-selective medium without hygromycin B, followed by re-inoculating the isolates onto selective plates containing hygromycin B. Antibiotic resistance and EGFP fluorescence were also examined.

Six out of seven transformants retained the ability to grow on hygromycin-B selective medium following passaging under non-selective conditions. Among these six resistant isolates, four exhibited normal EGFP fluorescence (Figures 10A–D), while two displayed obviously attenuated EGFP fluorescence signals despite retaining hygromycin-B resistance (Figures 10E,F). The remaining one resistant isolate lost EGFP-related fluorescence.

**FIGURE 10:**
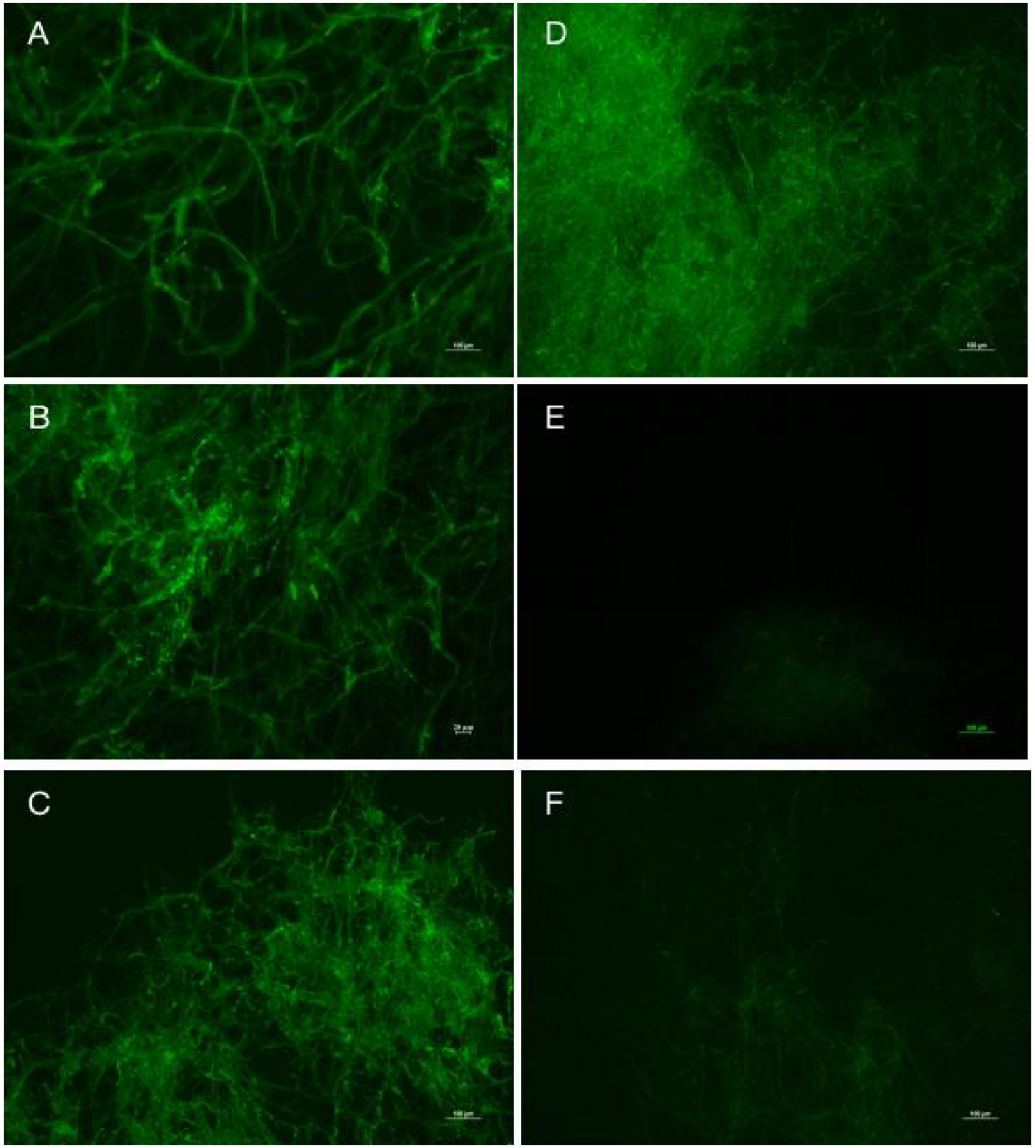
Fluorescence of transformants growing normally on selective medium after five serial subcultures. **(A–D)** Transformants with normal EGFP fluorescence. **(E–F)** Transformants with attenuated EGFP fluorescence.

### 3.9 Verification of pathogenicity of fluorescent strains

To evaluate whether EGFP expression affects pathogenicity, fluorescent transformant and wild-type strains were symmetrically inoculated onto wild apricot leaves, and disease development was monitored over a 12-day period. The temporal progression of lesion development was largely comparable between the two inoculated groups. Visible necrotic lesions were observed on both wild-type (WT)- and transformant-inoculated leaves at 2 days post-inoculation (Figure 11), and lesions gradually expanded into typical necrotic spots thereafter. Lesion diameter and chlorotic margins increased steadily from 4 d to 12 d post-inoculation, with no statistically significant differences between WT and transformant at all sampled time points (Figure 12). Disease symptoms peaked at 12 d post-inoculation, accompanied by complete necrosis and shedding of the inoculated leaf tissues. As the negative control, CK groups inoculated with sterile PDA showed no disease symptoms throughout the experiment, confirming that the observed necrotic lesions were caused by the inoculated fungal strains.

**FIGURE 11:**
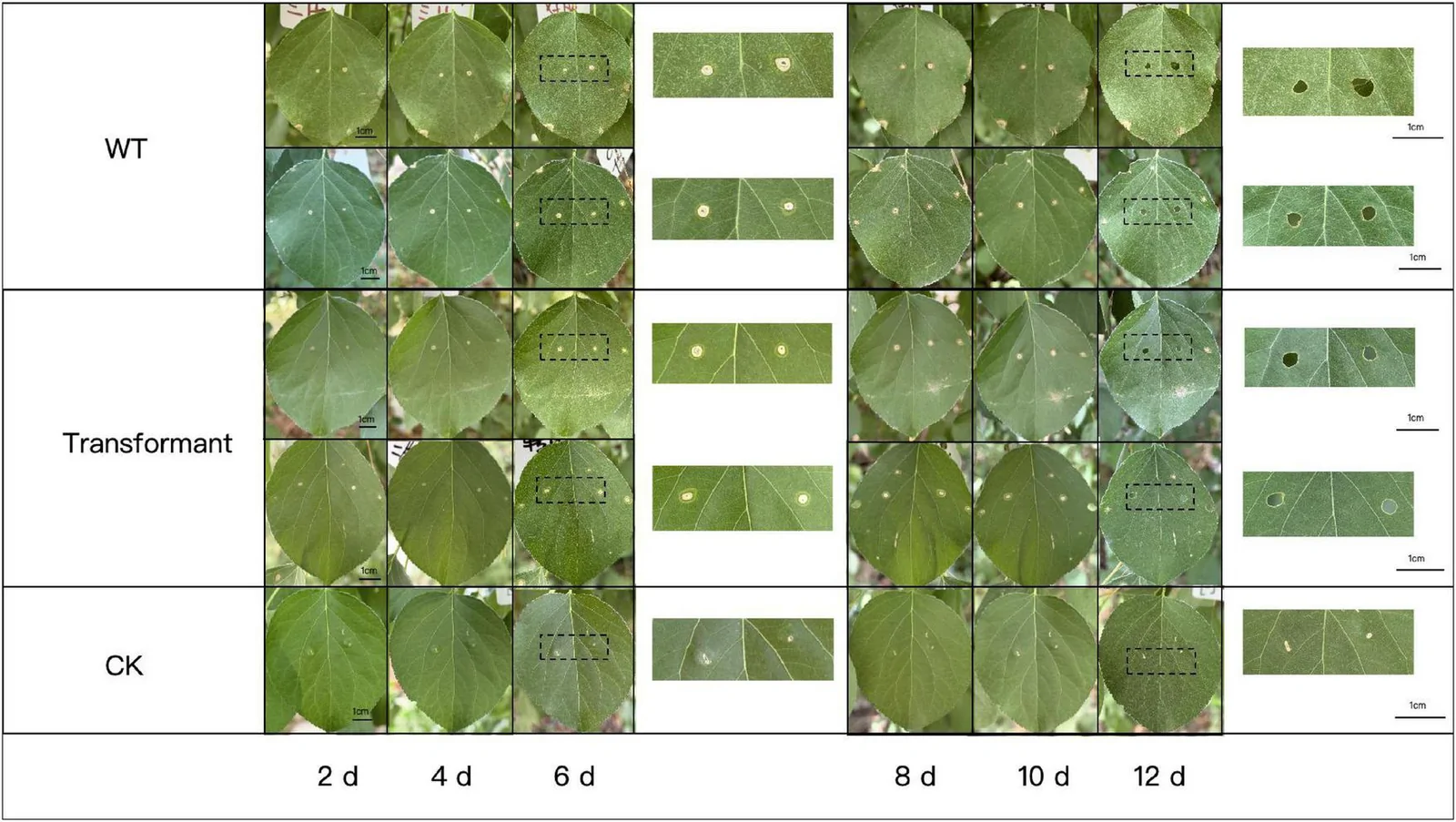
Lesion phenotypes on wild apricot leaves after inoculation with *Wilsonomyces carpophilus*. Rows represent wild-type (WT), fungal transformant, and PDA-only inoculation control (CK). Leaves were photographed at 2, 4, 6, 8, 10, and 12 days post-inoculation (d). Dashed boxes mark representative lesions, and magnified views of the marked lesions are displayed on the right-hand side of each panel. Scale bars represent 1 cm for whole-leaf images and 1 cm for magnified-lesion panels. n = 5 biological replicates for each treatment at each time point.

**FIGURE 12:**
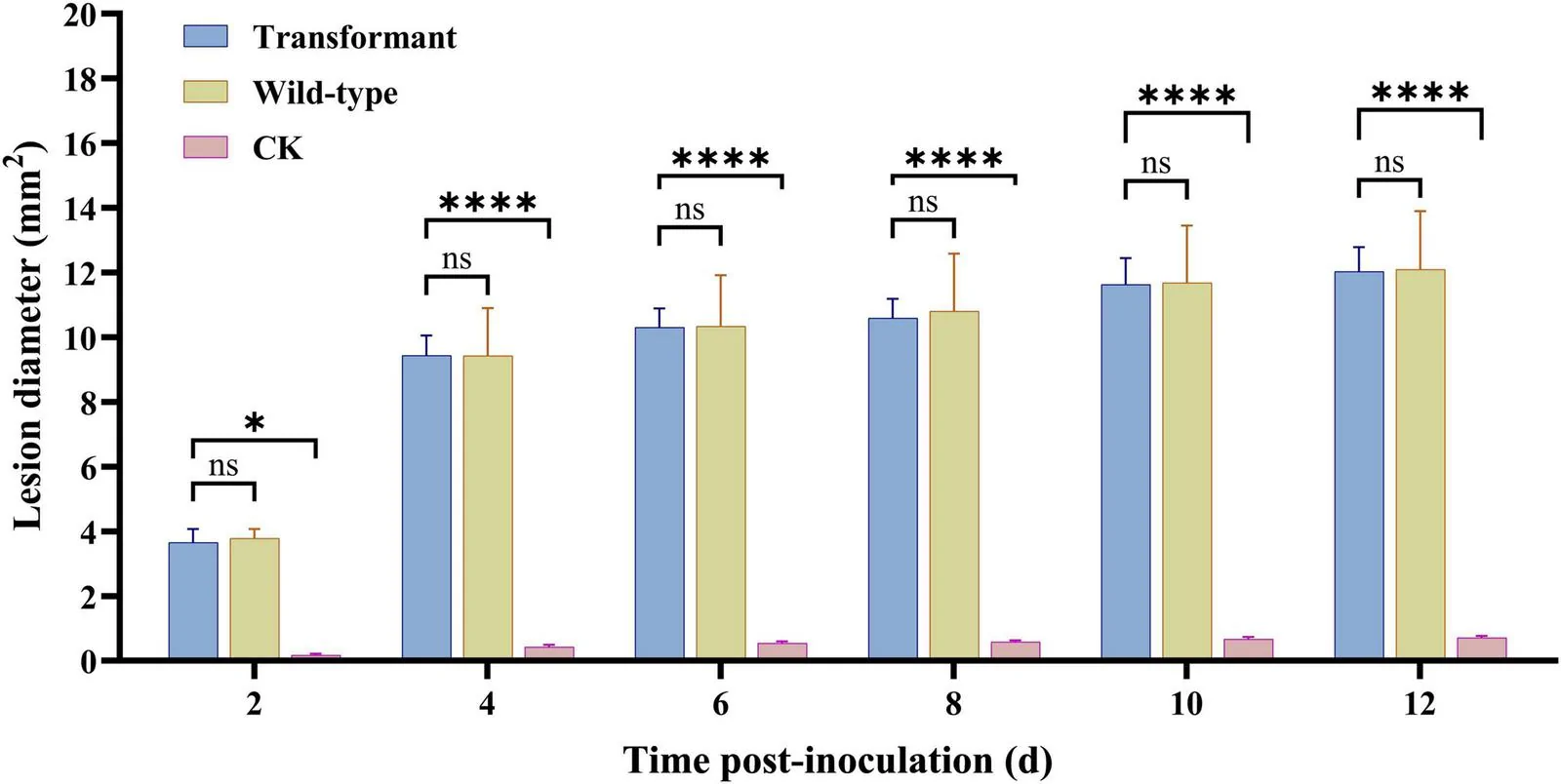
Measurement of lesion diameter on wild apricot leaves inoculated with *Wilsonomyces carpophilus*. Lesion diameters were measured at 2, 4, 6, 8, 10, and 12 days post-inoculation. Bars represent mean ± standard error of the mean (SEM). Transformant, fungal transformant; Wild-type, wild-type strain; CK, PDA-only inoculation control. Statistical analyses were performed using one-way ANOVA followed by Tukey’s multiple-comparison test. ns, no significant difference; *P < 0.05; ****P < 0.0001.

Quantitative lesion measurements were performed using ImageJ-Fiji software, and statistical analysis was conducted via *t*-test. No significant differences were detected in final lesion area (*p* > 0.05) (Figure 13A) and lesion expansion rate (*p* > 0.05) (Figure 13B) between fluorescent transformant and wild-type strain. These results demonstrated that *egfp* expression does not alter the pathogenicity of *W. carpophilus*.

**FIGURE 13:**
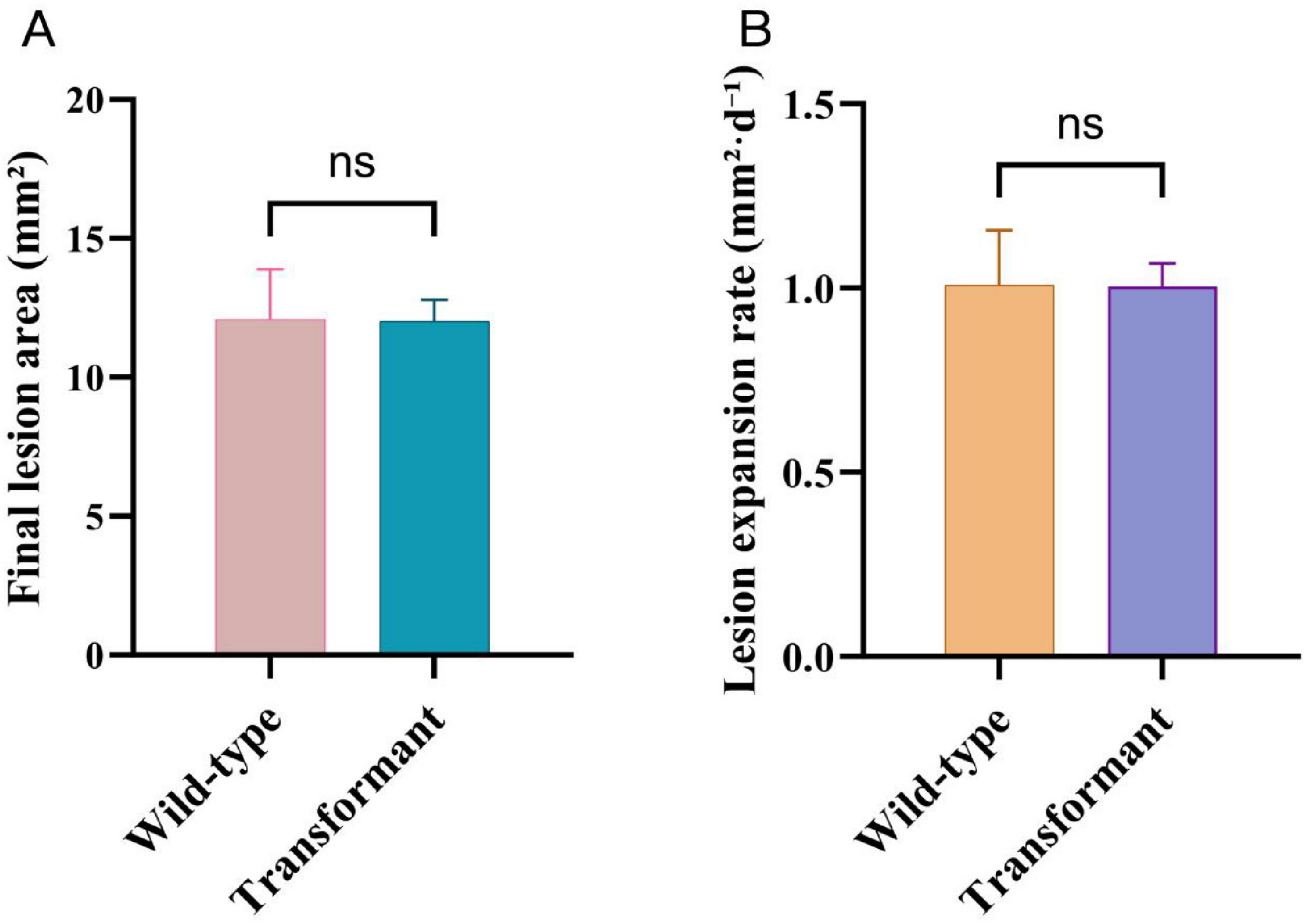
Final lesion area and lesion expansion rate of wild apricot leaves inoculated with *Wilsonomyces carpophilus*. **(A)** Final lesion area at 12 days post-inoculation. **(B)** Lesion expansion rate calculated over the infection period. Bars represent mean ± standard error of the mean (SEM). Wild-type, wild-type strain; Transformant, fungal transformant. ns, no significant difference.

### 3.10 Summary of optimized transformation conditions

The optimized conditions for protoplast preparation and PEG-mediated transformation of *W. carpophilus* strain G052 5 m2 are summarized in Table 2. The selected protocol used 24-h-old mycelia, enzymatic digestion with 20 g/L Driselase and 25 g/L Snailase for 5 h, and 1.2 mol/L NaCl as the osmotic stabilizer. These conditions produced the highest protoplast yield among the tested treatments and enabled the recovery of PCR-positive and EGFP-expressing transformants. No significant morphological or vegetative growth differences were observed between wild-type and transformants (*p* > 0.05). In addition, pathogenicity assays on wild apricot leaves revealed comparable lesion development dynamics, final lesion area and lesion expansion rate between wild-type and EGFP-tagged transformants across all monitored time points *(p* > 0.05), indicating that *egfp* integration did not impair vegetative growth or pathogenicity of *W. carpophilus*.

## 4 Discussion

### 4.1 Significance of establishing a transformation system for *Wilsonomyces carpophilus*

Genetic transformation systems are essential tools for studying gene function, pathogenicity mechanisms, and host–pathogen interactions in filamentous fungi. For *Wilsonomyces carpophilus*, the establishment of such a system is particularly important because this pathogen causes shot-hole disease in stone fruit trees and has been associated with severe disease symptoms in wild apricot populations in the Tianshan wild fruit forests. Although previous studies have addressed disease occurrence, biological characteristics, and control measures, molecular studies of this pathogen remain limited. The lack of an efficient transformation system has been a major obstacle to functional genetic analysis in *W. carpophilus*. In this study, we established a PEG-mediated protoplast transformation system for *W. carpophilus* strain G052 5 m2, providing a technical basis for future molecular studies of this pathogen.

### 4.2 Enzyme composition and mycelial age influence protoplast yield

The preparation of viable protoplasts is a critical step in PEG-mediated transformation. In filamentous fungi, protoplast release depends strongly on the composition of the fungal cell wall, the enzyme mixture used for digestion, and the physiological state of the mycelium. Because fungal cell wall composition differs among taxa and even among strains, transformation protocols developed for one species are not always suitable for another (Flor-Parra et al., 2014; Kang et al., 2020).

In *W. carpophilus*, the binary mixture of Driselase and Snailase yielded far more protoplasts than Snailase alone, whereas supplementing this cocktail with Lysing Enzymes conferred no further improvement. Fungal cell walls are predominantly composed of β-glucans, chitin, and mannoproteins; Driselase contains potent β–1,3-glucanase activity, and Snailase provides chitinase and protease activities, which together target the major structural polymers of the *W. carpophilus* cell wall. The lack of benefit from adding Lysing Enzymes suggests that additional glycosidase activities are redundant for degrading the cell-wall architecture of this species, rather than indicating insufficient enzyme loading. This highlights that higher-complexity enzyme cocktails do not always produce superior protoplast yields, and enzyme matching to species-specific cell-wall composition is more important.

Comparable species-specific responses have been reported in other filamentous fungi. For example, Driselase- and Lysing Enzyme-based combinations have been used for protoplast preparation in *Cytospora chrysosperma* and *Valsa mali*, whereas other fungi require different enzyme compositions and digestion times (Gao et al., 2011; Liu et al., 2017; Li et al., 2013; Lu et al., 2020). These differences support the need for species-specific optimization. The enzyme combination identified here provides a practical basis for reproducible protoplast preparation in *W. carpophilus*.

Mycelial physiological status is another key determinant of protoplast release. The lowest yield was obtained from 8-h-old cultures, whereas 24-h-old mycelia produced higher yields and were selected for subsequent experiments. In contrast, 42-h-old mycelia produced fewer protoplasts. This pattern indicates that young but sufficiently developed mycelia are more suitable for enzymatic hydrolysis in *W. carpophilus*. Very young cultures may not provide enough biomass, whereas older mycelia may develop thicker or more resistant cell walls, which confer higher resistance to enzymatic hydrolysis (Jin et al., 2020). 24-h-old culture represents a physiological sweet spot: sufficient vegetative biomass has accumulated, yet cell-wall components have not undergone extensive cross-linking or thickening. However, the optimal culture duration differs among fungi; for example, 42 h of shaking culture is suitable for *Botryosphaeria dothidea*, whereas several *Pleurotus* species require longer mycelial culture periods for efficient protoplast release (Chen et al., 2014; Eyini et al., 2006). Consequently, growth duration must be tuned alongside enzyme conditions for each target fungus.

### 4.3 Osmotic stabilizer selection improves protoplast recovery

Following cell-wall removal, protoplast membranes become fully exposed and are extremely susceptible to osmotic lysis; therefore, the osmotic stabilizer not only maintains turgor but also influences plasma-membrane integrity and post-digestion viability. In our assay, 1.2 mol/L NaCl supported significantly higher protoplast yields compared with 1.2 mol/L KCl under identical enzyme-digestion conditions.

The performance of osmotic stabilizers is species-specific and cannot be generalized across plant-pathogenic fungi. NaCl works efficiently for Gaeumannomyces graminis and some Penicillium species, whereas KCl is preferred for V. mali and C. chrysosperma (Chen et al., 2001; Gao et al., 2011; Liu et al., 2017). Beyond simple osmotic pressure maintenance, monovalent cations exert differential effects on fungal plasma-membrane stability and residual cell-wall fragment aggregation. For *W. carpophilus*, Na^+^ appears to better preserve membrane integrity during and after enzymatic digestion compared with K^+^, possibly by modulating surface-charge properties of membrane lipids or residual cell-wall polysaccharides. This observation emphasizes that osmotic stabilizer should not be chosen only on the basis of osmolarity; ion identity also matters, and stabilizer screening must be performed together with enzyme composition, digestion time, and mycelial age. Under our experimental setup, 1.2 mol/L NaCl provided the most suitable osmotic environment for protoplast preparation from *W. carpophilus* strain G052 5 m2.

### 4.4 Hygromycin B selection

An effective selection system is a prerequisite to separate genuine transformants from non-transformed background colonies following PEG-mediated transformation. Hygromycin B is a dominant selectable marker widely exploited in filamentous fungi, where expression of the hyg gene enables transformants to survive antibiotic pressure.

In this study, *W. carpophilus* strain G052 5 m2 exhibited high intrinsic sensitivity to hygromycin B, with 10 μg/mL completely suppressing wild-type growth. This working concentration is markedly lower than those applied for many other plant-pathogenic fungi (Robinson and Sharon, 1999; Zhang et al., 2011; Liu et al., 2017; Yan et al., 2021). Such pronounced inter-species divergence in hygromycin B susceptibility may stem from multiple factors. First, inherent differences in cell-wall composition and membrane permeability among fungal species can alter antibiotic uptake and intracellular accumulation. Second, species-specific drug-efflux capacity may contribute to distinct tolerance thresholds. The unusually high hygromycin B sensitivity of *W. carpophilus* highlights that selection pressure cannot be directly adopted from closely related taxa; empirical determination is indispensable for establishing an efficient screening system for each new fungal species.

### 4.5 Transformant validation and elimination of false positives

Our optimized experimental procedure enabled PEG-mediated delivery of the PCT74-EGFP construct into *W. carpophilus* protoplasts, yielding stable transformants verified by PCR amplification and EGFP fluorescence detection. The transformation efficiency obtained in this study was comparable to or slightly lower than that of well-established fungal transformation protocols. Variations in transformation efficiency across different fungal species are generally attributed to differences in protoplast activity, cell-wall regeneration capacity, and the intrinsic susceptibility of strains to exogenous DNA uptake. Given that no PEG-mediated protoplast transformation system has been previously reported for *W. carpophilus*, the current transformation efficiency can basically meet the experimental requirements for routine gene-function validation of this pathogen, yet further improvement is still needed. Nevertheless, the recovered transformants exhibited consistent molecular profiles and fluorescent phenotypes, confirming the feasibility of this transformation system.

It should be noted that a proportion of hygromycin B-resistant colonies were false-positives, a common drawback in PEG-protoplast transformation caused by heterokaryon formation. Notably, two isolates retained stable hygromycin-B resistance after three successive rounds of antibiotic screening but failed to generate the expected target PCR amplicon. Two plausible mechanisms may account for this observation. First, heterokaryon formation, a frequent event during filamentous-fungal transformation that can lead to unstable phenotypes or PCR-detection failure. In heterokaryons, transformed and wild-type nuclei coexist within the same mycelium; nuclei carrying the hygromycin-resistance cassette sustain antibiotic tolerance but may be present at too-low abundance to be detectable by PCR (Hamada et al., 1997; Guo and Cooley, 1990). Second, partial or truncated plasmid integration, in which only the hygromycin-resistance cassette is integrated into the genome while the target fragment for PCR amplification is lost during non-homologous end-joining-mediated integration (Asch and Kinsey, 1992; Vaillancourt and Hanau, 1994; Watson et al., 2008). Therefore, multi-round antibiotic purification combined with molecular verification is essential to filter out non-transformed nuclei and obtain pure mutant strains. In general, our protocol follows the canonical technical framework for PEG-mediated transformation in filamentous fungi (Liu and Friesen, 2012), and provides a foundational tool for future gene-function research on *W. carpophilus*.

Mitotic stability assays revealed that some transformants maintained hygromycin-B resistance yet exhibited attenuated or lost EGFP fluorescence after serial subculture. This phenomenon may result from heterokaryon formation or partial silencing of the integrated expression cassette. Although phenotypic tests provided preliminary evidence for transformant stability, definitive confirmation of transgene integration still requires Southern blotting, which is proposed for future investigations.

### 4.6 Complementarity of PEG-mediated transformation and ATMT for *W.* carpophilus

Rasool et al. (2020) previously reported an Agrobacterium tumefaciens-mediated transformation (ATMT) system for *W. carpophilus* (syn. *Thyrostroma carpophilum*), in which conidial spores served as transformation recipients. Their ATMT protocol yielded transformants predominantly with single-copy T-DNA insertions and enabled construction of a large insertional mutant library for pathogenicity-related gene screening. Despite these merits, ATMT strictly relies on conidia as starting material. The isolate G052 5 m2 used in the present study displays delayed and slow sporulation in culture. For this slow-sporulating isolate, our PEG-mediated protoplast transformation uses mycelia as starting material. The mycelium is available for protoplast preparation after only 3-day cultivation, which greatly cuts down sample-preparation duration and improves overall experimental efficiency relative to spore-dependent ATMT. Nevertheless, PEG-mediated transformation requires tedious protoplast isolation and regeneration procedures, and multi-copy integration is more common, requiring further molecular validation for transformants. Therefore, these two transformation systems are complementary and can be selected according to experimental purposes and fungal strain characteristics.

### 4.7 Limitations and future applications

Although this study established a workable transformation system for *W. carpophilus*, several aspects remain to be improved in future investigations. First, protoplast viability should be assessed via PI/FDA staining to better characterize the physiological status of enzymatically-prepared protoplasts. Second, protoplast regeneration efficiency should be quantitatively determined, which represents a critical parameter for evaluating transformation performance. Finally, PCR can only confirm the presence of exogenous genes in transformants and cannot verify stable genomic integration of foreign fragments. Further experiments including Southern blotting and qPCR-based copy-number analysis are therefore required to validate the integration status of exogenous DNA.

Overall, this study reports the first PEG-mediated protoplast transformation system for *W. carpophilus*. Using 24-h-old mycelia as starting material, protoplasts were generated with 20 g/L Driselase plus 25 g/L Snailase in 1.2 mol/L NaCl osmotic stabilizer, and the PCT74-EGFP plasmid was successfully introduced via PEG-mediated transformation, yielding a transformation efficiency of 1.75 transformants per μg DNA. This optimized system provides a reliable molecular tool for future research on gene function, pathogenic mechanisms, and host-pathogen interactions of this important stone-fruit pathogen.

## 5 Conclusion

This study established a PEG-mediated protoplast transformation system for *Wilsonomyces carpophilus* strain G052 5 m2, a shot-hole disease pathogen associated with wild apricot in the Tianshan wild fruit forests. The optimized protocol used 24-h-old mycelia, enzymatic digestion with 20 g/L Driselase and 25 g/L Snailase for 5 h, and 1.2 mol/L NaCl as the osmotic stabilizer, yielding up to 4.13 × 10^6^ protoplasts/mL. The successful introduction of the PCT74-EGFP plasmid was verified by antibiotic selection, PCR amplification, and EGFP fluorescence observation. No significant differences in colony morphology or growth rate were observed between transformants and the wild type. This confirms the feasibility of the transformation system. As the first reported genetic transformation protocol for *W. carpophilus*, this work provides a practical molecular tool for future studies on gene function, pathogenicity mechanisms, and host–pathogen interactions in this important stone fruit pathogen. Nevertheless, this transformation system still suffers from limitations including moderate transformation efficiency. Future studies will exploit this system for targeted gene disruption, CRISPR/Cas9-mediated editing, and functional characterization of pathogenicity-related genes to deepen our understanding of this pathogen.

## Data Availability

The original contributions presented in the study are included in the article/supplementary material, further inquiries can be directed to the corresponding author.
